# A Complex Pattern of Gene Expression in Tissue Affected by Viperid Snake Envenoming: The Emerging Role of Autophagy-Related Genes

**DOI:** 10.3390/biom14030278

**Published:** 2024-02-26

**Authors:** Ana Karina de Oliveira, Alexandra Rucavado, Teresa Escalante, José María Gutiérrez, Jay W. Fox

**Affiliations:** 1 Department of Pathology, University of Virginia School of Medicine, Charlottesville, VA 22908, USA; ak4yj@virginia.edu; 2 Instituto Clodomiro Picado, Facultad de Microbiología, Universidad de Costa Rica, San José 11501, Costa Rica; alexandra.rucavado@ucr.ac.cr (A.R.); teresa.escalante@ucr.ac.cr (T.E.); jose.gutierrez@ucr.ac.cr (J.M.G.); 3 Department of Microbiology, Immunology and Cancer Biology, University of Virginia School of Medicine, Charlottesville, VA 22908, USA

**Keywords:** snake venom, *Bothrops asper*, *Daboia russelii*, gene expression, myonecrosis, Nanostring, extracellular matrix, autophagy, immune microenvironment

## Abstract

Viperid snake venoms induce severe tissue damage, characterized by the direct toxic action of venom components, i.e., phospholipases A_2_ (PLA_2_s) and metalloproteinases (SVMPs), concomitantly with the onset of endogenous inflammatory processes, in an intricate scenario of tissue alterations. Understanding the expression of relevant genes in muscle tissue will provide valuable insights into the undergoing pathological and inflammatory processes. In this study, we have used the Nanostring technology to evaluate the patterns of gene expression in mouse skeletal muscle 1 h, 6 h, and 24 h after injection of the venoms of *Bothrops asper* and *Daboia russelii*, two medically relevant species in Latin America and Asia, respectively, with somewhat different clinical manifestations. The dose of venoms injected (30 µg) induced local pathological effects and inflammation in muscle tissue. We focused our analysis on genes related to extracellular matrix (ECM) metabolism, immune system, programmed cell death, and autophagy. The results revealed a complex pattern of expression of genes. Regarding ECM metabolism and regulation, up-regulated genes included proteinase inhibitor Serpine 1, thrombospondin 1, collagens 1A1 and 4A1 (at 1 h in the case of *B. asper*), TIMP1, MMP-3 (at 24 h), and lysil oxidase (LOX). In contrast, collagen chains 5A3 and 5A1 were down-regulated, especially at 6 h. Transforming growth factor β (TGF-β) and several genes related to myofibroblast regulation were also up-regulated, which might be related to the development of fibrosis. Several genes related to cytokine and chemokine synthesis and regulation and NFκB signaling were also up-regulated. Our observations show a variable expression of genes associated with programmed cell death and autophagy, thus revealing a hitherto unknown role of autophagy in tissue affected by snake venoms. These results provide clues to understanding the complex pattern of gene expression in tissue affected by viperid snake venoms, which likely impacts the final pathophysiology of damaged tissue in envenomings.

## 1. Introduction

Envenomings by viperid snakes are characterized by severe pathological effects at the anatomical site of venom injection, associated with tissue necrosis, hemorrhage, blistering, and edema [1,2]. Such effects are primarily caused by the direct action of tissue-damaging toxins, mostly phospholipases A_2_ (PLA_2_s) and metalloproteinases (SVMPs) [3,4,5]. Concomitantly, a complex inflammatory response develops in the affected tissues, characterized by the recruitment of inflammatory cells, i.e., neutrophils and macrophages, the activation of resident cells, and the synthesis of a plethora of inflammatory mediators and damage-associated molecular patterns (DAMPs) from damaged cells and extracellular matrix [6,7]. Such endogenous response contributes to edema and clearance of damaged tissue and sets the stage for reparative and regenerative processes. At the same time, local inflammation might potentiate some of the deleterious effects caused by the venom.

Understanding this altered tissue environment has been challenging because of its complexity, its dynamics over time, and its spatial heterogeneity in various regions of the affected tissue [8]. Several studies have quantified specific inflammatory mediators in tissues or blood in experimental models of viperid envenomings and have contributed to our understanding of these phenomena (see reviews by Teixeira et al. [6,9]). However, to fully grasp the complexity of this inflammatory scenario it is necessary to introduce novel analytical tools that allow the analysis of a vast number of tissue components and inflammatory mediators. This will provide novel clues on the genesis and dynamics of inflammatory and tissue reparative and regenerative processes. To this end, the Nanostring nCounter platform is one approach to provide novel clues by allowing the analysis of the expression of up to 800 genes simultaneously, without amplification, cDNA, or library preparation [10,11]. This technology uses direct, digital quantitation of mRNA transcripts by hybridization to color-barcode-specific probes in a high-sensitivity manner [10]. The ability of probes to hybridize a complementary region of 100 nucleotides long allows this technology to measure mRNA with low abundance and sub-optimal RNA quality. In this context, analyzing RNA extracted from formalin-fixed paraffin-embedded (FFPE) tissue samples allows us to investigate the correlation between mRNA expression and the histological alterations occurring in the tissue (spatial omics).

Over the past years, our group has focused our studies on several critical aspects of the pathological and inflammatory effects induced by the venoms of two viperid species, *Daboia russelii* and *Bothrops asper*, which are medically relevant snakes in Asia and Latin America, respectively, owing to the high number of cases they inflict and to the severity of clinical manifestations. These venoms inflict envenomings with some similar clinical features, including local tissue damage, hemorrhage, coagulopathy, and hemodynamic disturbances [12,13]. At the same time, there are notable differences in their actions since *D. russelii* envenomings are also characterized by systemic capillary leakage syndrome, high incidence of acute kidney injury, and, in some geographical locations, neurotoxicity [12], effects, which are largely absent in *B. asper* envenomings. Experimentally, these two venoms induce a different pattern of vasculotoxicity since *B. asper* venom causes strong hemorrhage in rodent models, while *D. russelii* venom induces less extensive hemorrhage but a conspicuous increase in vascular permeability, associated with hemoconcentration [14,15]. Furthermore, they show variations in the patterns of increments in some inflammatory mediators [16]. Lipidomic and metabolomic analyses of plasma from mice injected with these venoms demonstrated both similarities and differences [17]. However, there is a notorious lack of information on the patterns of expression of genes that might be involved in the events occurring in tissue injected with venoms.

To further explore and compare the effects of these two venoms in the muscle tissue of mice, in this study, we have employed the Nanostring nCounter technology using the mouse Fibrosis V2 panel to follow the mRNA expression of several markers associated with extracellular matrix synthesis and degradation, innate immune system changes, and cell death pathways. Our goal was to delineate the patterns of gene expression in envenomed tissue, which may explain some of the pathological effects of envenoming and reveal novel mediators and processes involved in tissue damage and repair. Our findings highlight similarities and differences between the action of these venoms and identify trends in the time course of the expression of these markers. They also allow the identification of tissue and inflammatory markers, which further our understanding of venom-induced pathology and inflammation. 

## 2. Materials and Methods

### 2.1. Venoms

The venom of *D. russelii* was purchased from Latoxan (Code L1132A; Lot: 015.051; Portes-lès-Valence, France). It is a pool of venom collected from several adult specimens from Pakistan. The venom of *B. asper* was provided by Instituto Clodomiro Picado, University of Costa Rica. It is a pool of venom from more than 20 adult specimens collected in the Pacific region of Costa Rica and kept at the Serpentarium of Instituto Clodomiro Picado. Venoms were freeze-dried and stored at −20 °C. They were dissolved in 0.12 M NaCl, 0.04 M phosphates, pH 7.2 (PBS) immediately before use.

### 2.2. Experimental Protocol

Groups of three mice of both sexes (CD-1 strain, 18–20 g) received an intramuscular injection in the right gastrocnemius of 30 µg venom of each species, dissolved in 50 µL PBS. Control mice were injected with 50 µL PBS only. At the time intervals of 1 h, 6 h, and 24 h following injection, groups of three mice were sacrificed by CO_2_ inhalation, and a sample of the injected gastrocnemius muscle was excised and added to 10% formalin solution in water. After 48 h fixation, routine processing of tissues was performed, followed by embedding in paraffin. Muscle tissue sections (4 µm thickness) were stained with hematoxylin and eosin or Masson’s trichrome for histological observation of tissue damage and inflammatory infiltrate. The protocols of experiments using mice were approved by the Institutional Committee for the Care and Use of Laboratory Animals (CICUA) of the University of Costa Rica (approval number CICUA-032-2020) and meet the International Guiding Principles for Biomedical Research Involving Animals (CIOMS). Mice were maintained in Tecniplast Eurostandard Type II 1264C cages (L 25 × W 40 × H 14 cm), six mice per cage. Animals were kept at 18–24 °C, 60–65% relative humidity, and a 12:12 h light–dark cycle.

### 2.3. RNA Extraction

A total of 21 blocks of formalin-fixed paraffin-embedded muscle mouse tissue (Control, *B. asper* (BA) BA-1 h, BA-6 h, BA-24 h, *D. russelii* (DR) DR-1 h, DR-6 h, DR-24 h) were used for RNA extraction. Four slices of 10 µm thickness from each selected block were deparaffinized using D-Limonene (Sigma, Kawasaki, Japan), digested with proteinase K, and isolated using RNeasy^®^ Plus Mini Kit QIAGEN, according to the manufacturer’s protocol. The final volume of extracted RNA was 14 µL. RNA concentration and purity were assessed using a NanoDrop instrument. Sample concentration was measured at 260 nm and 280 nm, and the ratio of optical density 260/280 and 260/230 were used to test for protein and phenol contamination, respectively. 

### 2.4. NanoString nCounter Analysis

Comparative analysis between samples collected from mice injected with *B. asper* and *D. russelii* venoms was conducted by Nanostring nCounter multiplex analysis [10] using the Mouse Fibrosis V2 panel (Supplementary Table S1). Nanostring probes are made with target-specific sequences and tag-specific sequences at 5′ and 3′ tailing ends (Supplementary Table S1). The RNA samples (100 ng) were incubated for 16 h at 65 °C in a hybridization buffer containing the CodeSet (reporter and capture probes). Hybridized samples were processed using the Prep Station using high sensitivity protocol, 3 h per 12-sample cartridge. The Prep Station purifies the RNA/probe complexes and places them in a cartridge where they are immobilized and aligned for data collection. Data acquisition was carried out in the NanoString nCounter Digital Analyzer with the ‘Max’ Field of View (FOV) setting to 555 images per sample in a 6 h scan per cartridge. Raw counts were normalized using the positive controls, and target genes were normalized to the five internal reference genes (Supplementary Table S2).

### 2.5. Data Analysis

NanoString gene expression profiling was conducted using the mouse nCounter Fibrosis V2 Panel (NanoString Technologies, Seattle, WA, USA), which contains probes specific to 760 endogenous genes and 10 housekeeping genes (ACAD9, ARMH3, CNOT10, GUSB, MTMR14, NOL7, NUBBP1, PGK1, PPIA, RPLP0) described in Supplementary Table S1.

Data were analyzed using ROSALIND (https://rosalind.bio/, accessed on 1 September 2023), with a hyper-scale cloud developed by ROSALIND, Inc. (San Diego, CA, USA), accessed on 5 March 2023. The quality control of samples was verified using spike-ins of positive and negative probes and housekeeping expression levels (Supplementary Figure S1). Normalization, fold changes, and *p*-values were calculated using criteria provided by NanoString. ROSALIND follows the nCounter Advanced Analysis protocol of dividing counts within a lane by the geometric mean of the normalizer probes from the same lane. The geNorm algorithm was used to select normalizer probes in the Bioconductor package NormqPCR, removing candidate housekeepers with the least stable expression relative to other candidates (Supplementary Table S2).

Fold changes and *p*-values were calculated using the “Fast” method, as described in the nCounter Advanced Analysis 2.0 User Manual (MAN-10030-03). Differential gene expression analysis was calculated using the generalized linear model (GLM) that was developed by NanoString for analysis of count data, assuming a negative binomial distribution. Adjusted *p*-values are calculated using the Benjamini-Hochberg False Discovery Rate (FDR) methodology (Supplementary Table S3).

### 2.6. Enrichment Pathway Analysis

The enrichment pathways were analyzed using the Gene Set Analysis (GSA) module from NanoString. GSA summarizes the change in regulation within each defined gene set relative to the baseline, as described in the manufacturer’s manual (MAN-10030-03). The values calculated are the Global Significance Score (also called Undirected Global Significance Score), which measures the overall differential expression of the selected gene set relative to selected patient populations, ignoring whether each gene is up- or down-regulated (Supplementary Table S4).

Another variant calculated was the Directed Global Significance scores, which measure the extent to which a given gene set is up- or down-regulated relative to a given covariate. It is calculated similarly to the undirected global significance score, but it takes the sign of the t-statistics into account (Supplementary Table S4).

## 3. Results and Discussion

### 3.1. Histopathological Observations

**Figure 1:**
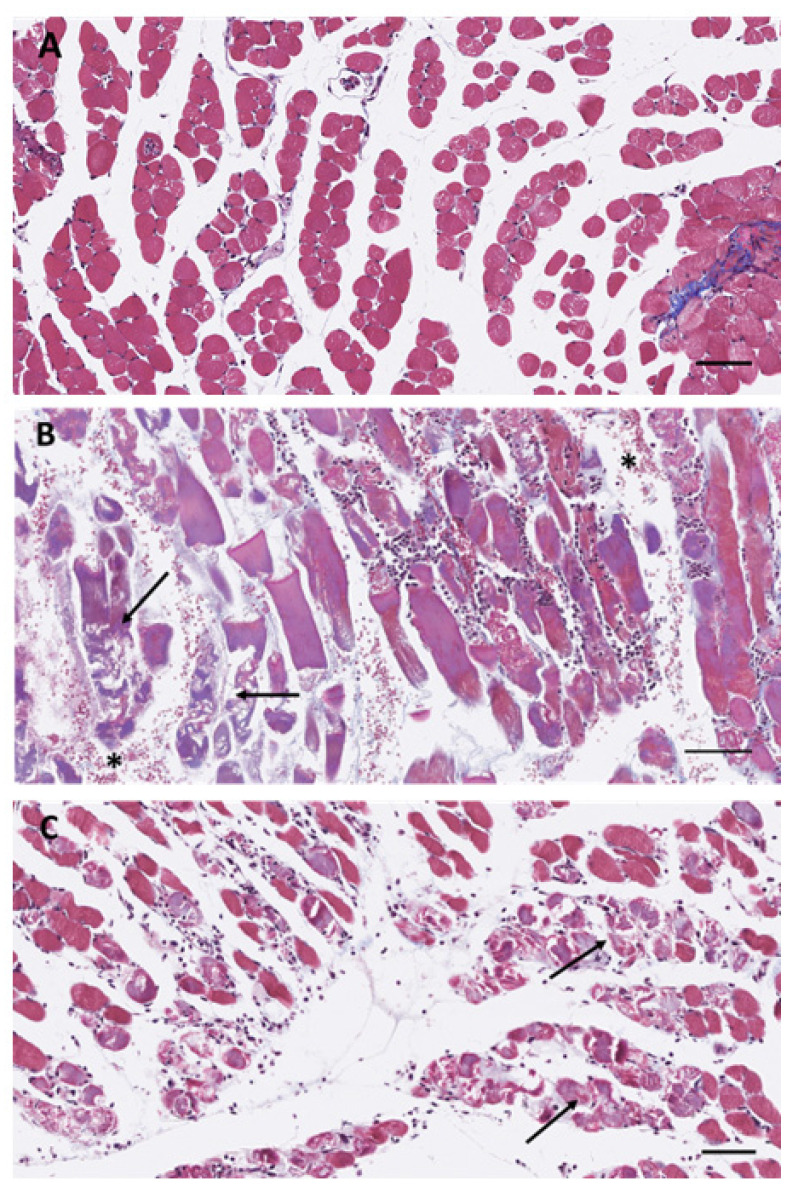
Light micrographs of sections of skeletal muscle from mice injected in the right gastrocnemius muscle with (**A**) PBS, (**B**) 30 µg *B. asper* venom dissolved in 50 µL PBS, or (**C**) 30 µg *D. russelii* venom dissolved in 50 µL PBS. Mice were sacrificed 24 h after injection, and the gastrocnemius muscles were excised, placed in formalin fixative, and routinely processed for embedding in paraffin (see Section 2 for details). Muscle tissue sections (4 µm thickness) were stained with Mallory’s trichrome for histological observation of tissue damage and inflammatory infiltrate. Both venoms induced muscle fiber necrosis (arrows), but only *B. asper* venom induced hemorrhage, as evidenced by the presence of erythrocytes in the interstitial space (*). An inflammatory infiltrate of leukocytes is present in envenomed tissue. Bars correspond to 100 µm.

The dose of venoms used in this study (30 µg) was selected on the basis of previous studies and corresponds to a dose that induces overt pathological and inflammatory effects in the muscle tissue of mice [14,16]. In agreement with these previous reports [14,16], histological analysis of muscle tissue injected with venoms of *B. asper* and *D. russelii* revealed a different pattern of local tissue damage. *B. asper* venom induced myonecrosis and hemorrhage, evidenced by the presence of hypercontracted muscle fibers and abundant erythrocytes in the interstitial space. In contrast, tissue from mice injected with *D. russelii* venom showed muscle fiber necrosis, but hemorrhage was largely absent. In both cases, an inflammatory infiltrate was observed in the damaged tissue (Figure 1) 

### 3.2. ECM Synthesis, Degradation, and Modification

**Figure 2:**
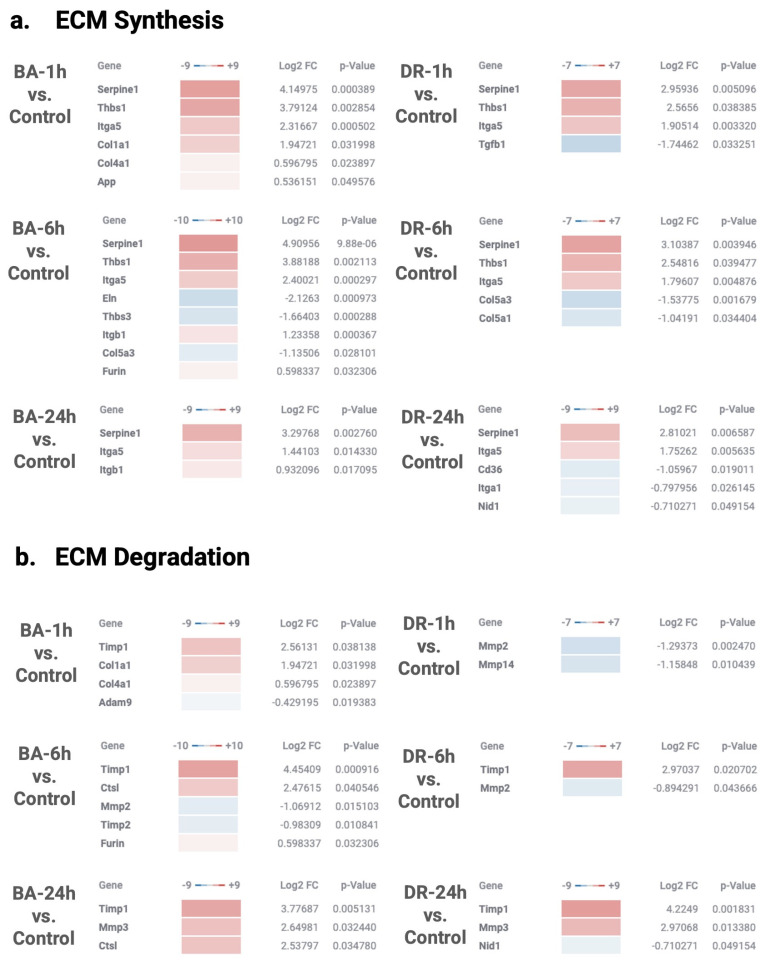

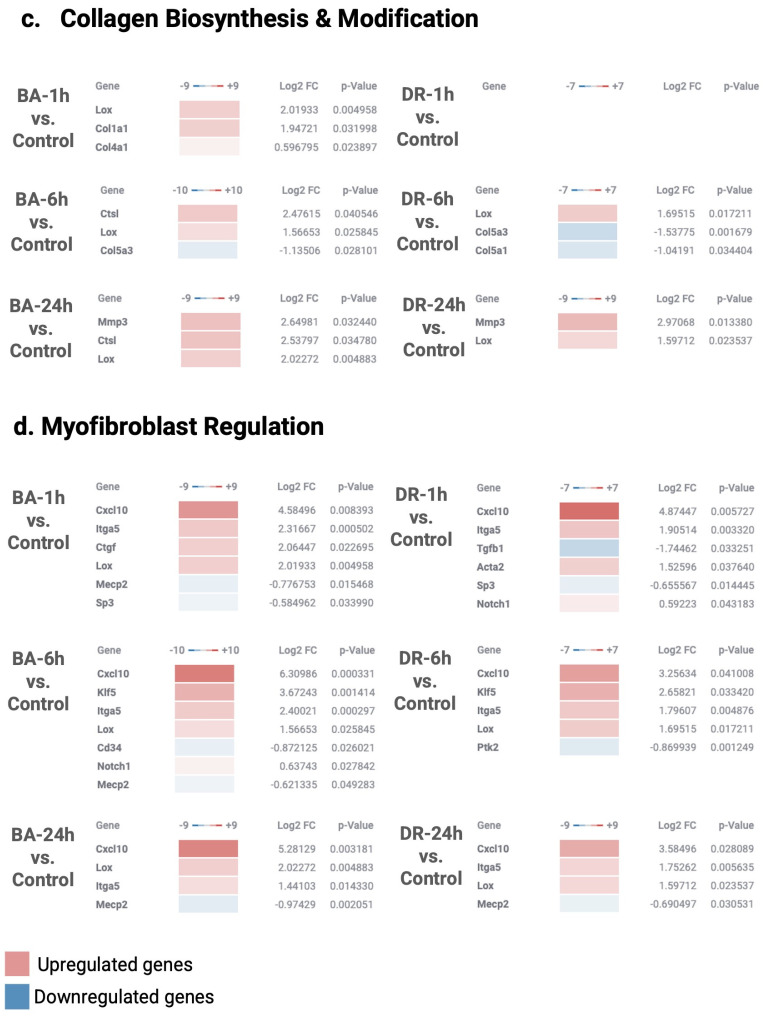
NanoString GSA pathway analysis of extracellular matrix synthesis (**a**), extracellular matrix degradation (**b**), collagen biosynthesis and modification (**c**), and myofibroblast regulation (**d**) genes alterations in muscle tissue injected with venoms of *B. asper* and *D. russelii*, as compared to controls injected with PBS. The Directed Global Significance scores are represented by up-regulated (red) and down-regulated (blue) genes at different time points in relation to the control samples. The *p*-value and Log2 FoldChange are shown to the right. Global Significance Score > 1.0. Graphic created in the Rosalind software (https://rosalind.bio/ version 3.38.6.1, accessed on 5 March 2023).

**Figure 3:**
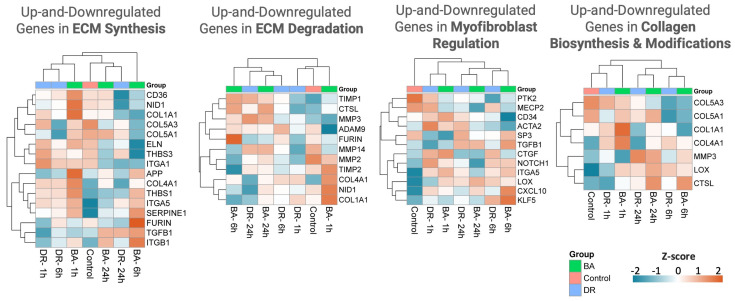
Heat maps of the extracellular matrix and myofibroblast regulation genes expression in muscle tissue injected with PBS, *B. asper* venom, or *D. russelii* venom. Heat maps were made using Log2 of transcript counts of the up- and down-regulated significant genes in each pathway using Gene Set Analysis (GSA). Each row represents the gene monitoring over time after venom injection. Euclidean distance and complete linkage methods were applied. Hierarchical clustering was performed with SRPLOT (accessed on 1 January 2023 at https://www.bioinformatics.com.cn).

A variable pattern of expression of genes associated with ECM synthesis, degradation, and modification was observed at the three time intervals in samples from muscle tissue injected with *B. asper* and *D. russelii* venoms, with quantitative differences in the fold change expression between venoms and between time intervals (Figure 2 and Figure 3). Up-regulated genes, as compared to control samples, included the proteinase inhibitor Serpine 1, thrombospondin 1, collagens 1A1 and 4A1 (at 1 h in the case of *B. asper*), TIMP1, MMP-3 (at 24 h), lysil oxidase (LOX), and integrin subunit α5, among others. As a general trend, changes were more pronounced in samples collected from *B. asper*-injected mice, especially at 1 and 6 h (Figure 2 and Figure 3). In contrast, genes coding for MMP-2 and collagen 5A3 and 5A1 were down-regulated, especially at 6 h, compared to controls injected with PBS only (Figure 2 and Figure 3). 

These findings reveal a variable pattern of gene regulation, with some genes coding for fibrillar (collagen I) and basement membrane (collagen IV) collagens being up-regulated, while chains of collagen V were down-regulated. Expression of MMPs also evidenced up- and down-regulation, whereas two proteinase inhibitors were up-regulated, indicating a process of modulation of ECM degradation. Fragments of several serpins and TIMPS have been described in exudates collected from mice injected with venom and tissue-damaging toxins from *B. asper* [18,19], implying a control over ECM degradation by these proteinase inhibitors. Our observations reveal a variable pattern of expression of genes coding for ECM-associated proteins within the previously described pattern of ECM degradation, synthesis, and modulation [18,19,20,21,22]. The consequences in the overall tissue damage of this differential expression of MMPs and ECM components remain to be investigated. Despite some qualitatively similar patterns observed in gene expression between the two venoms, tissue from *B. asper*-injected venom showed a higher quantitative expression of most of these proteins. This is likely related to the fact that *B. asper* venom has higher proteolytic and hemorrhagic activities than *D. russelii* venom, in agreement with the higher content of SVMPs in *B. asper* venom [23,24,25], thus causing more extensive ECM degradation and turnover. It is necessary to further investigate the actual degradation patterns of ECM proteins in these in vivo models, to assess which components are degraded at different time intervals. Our findings suggest a higher ECM turnover in muscle injected with *B. asper* venom. 

**Figure 4:**
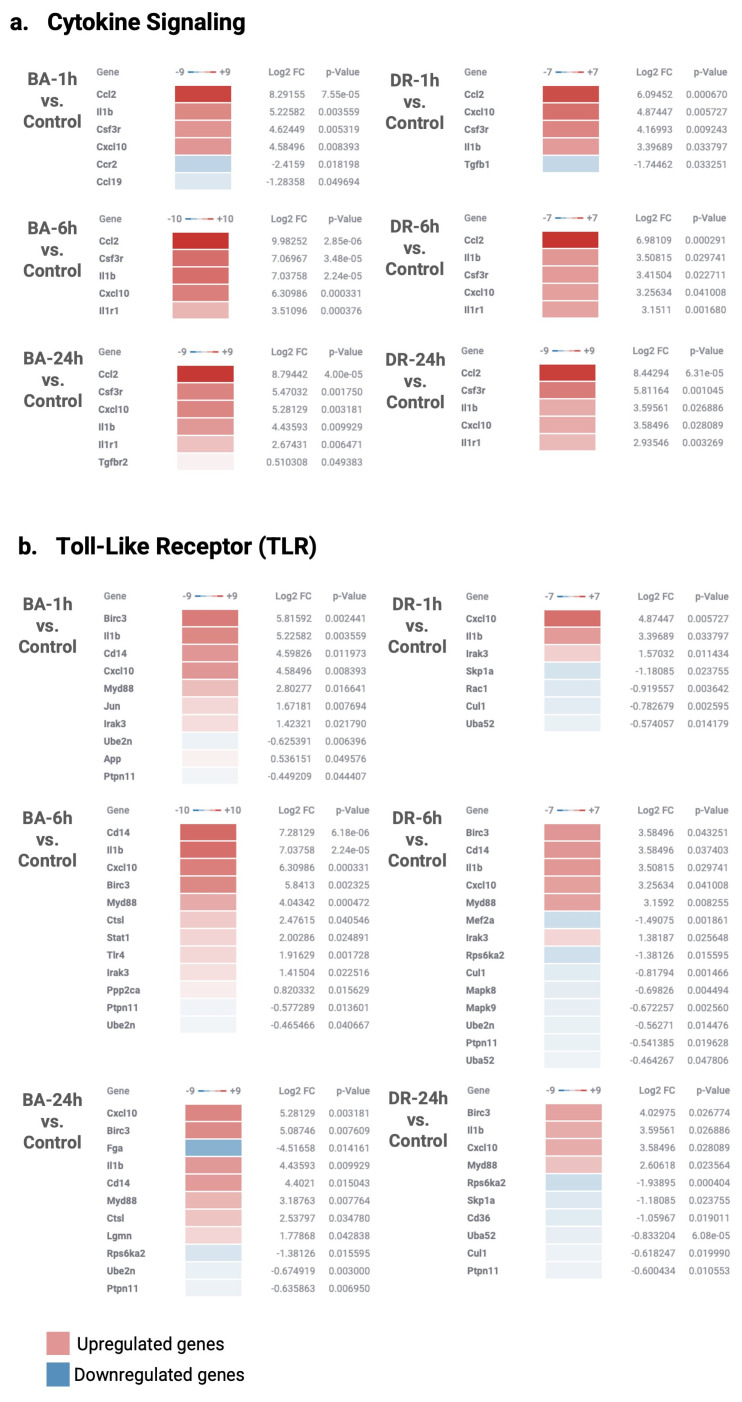

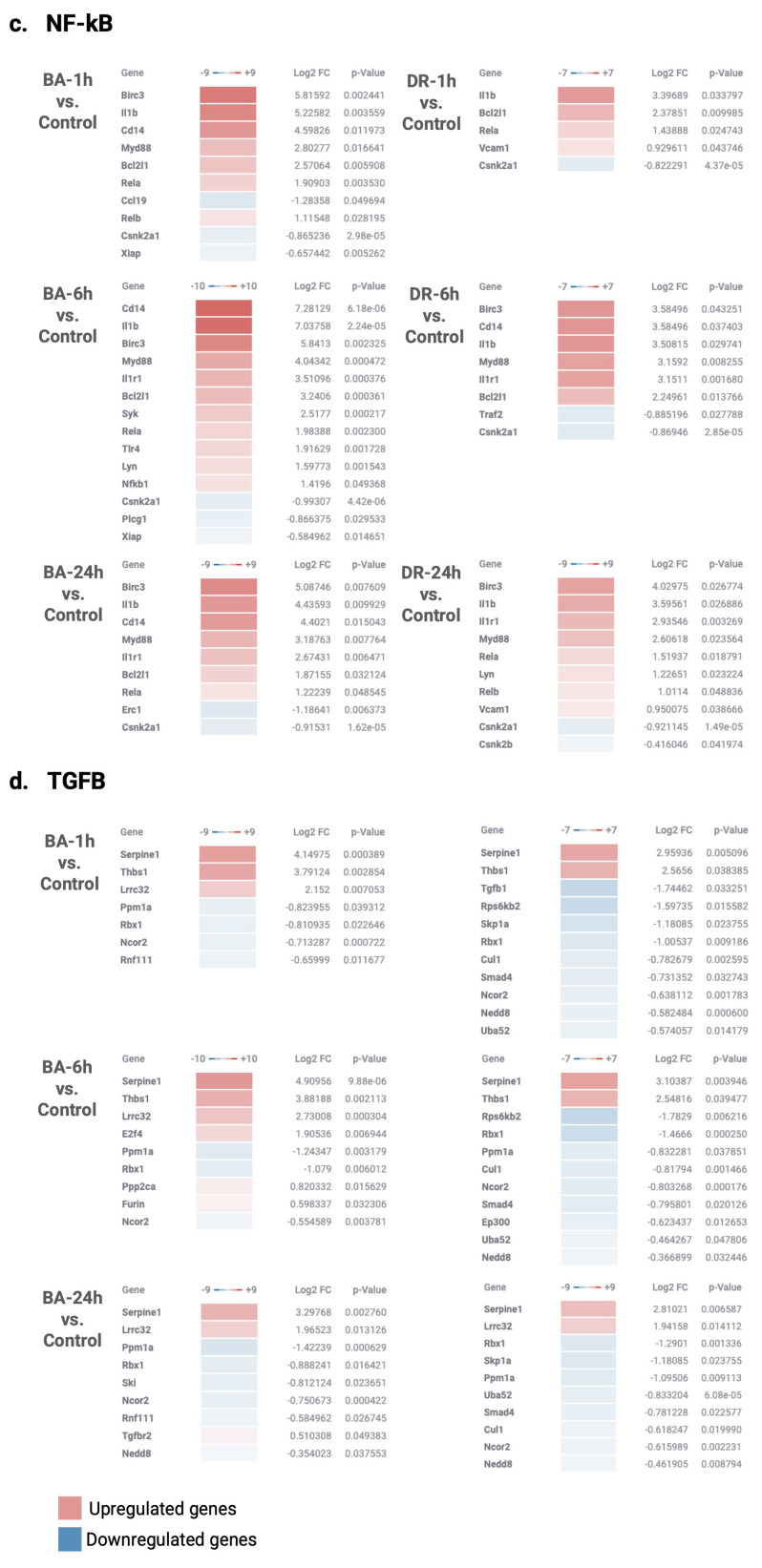
NanoString GSA pathway analysis of cytokine signaling (**a**), Toll-like receptors (**b**), NK-κB (**c**), and TGFβ (**d**) genes in muscle tissue injected with venoms of *B. asper* and *D. russelii*, as compared to controls injected with PBS. The Directed Global Significance scores are represented by up-regulated (red) and down-regulated (blue) genes at different time points in relation to the control samples. The *p*-value and Log2 FoldChange are shown to the right. Global Significance Score > 1.0. Graphic created in the Rosalind software (https://rosalind.bio/ version 3.38.6.1, accessed on 5 March 2023).

**Figure 5:**
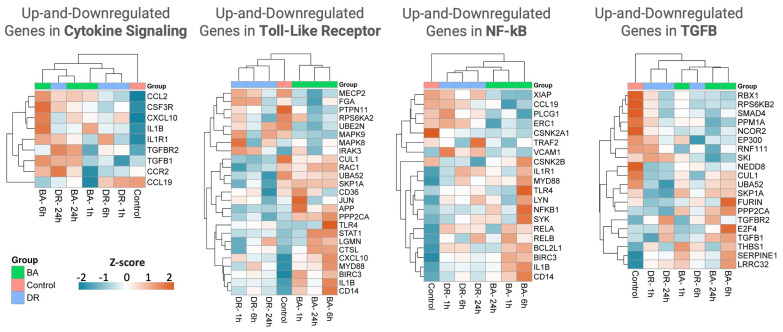
Heat maps of the cytokine signaling, Toll-like receptors, NK-κB, and TGFβgenes alterations in samples from mice injected with either PBS (control), *B. asper,* or *D. russelii* venoms. The heat maps were made using Log2 of transcript counts of the up- and down-regulated significant genes in each pathway using the Gene Set Analysis (GSA). Each row represents the gene monitoring over time after venom injection. Euclidean distance and complete linkage methods were applied. Hierarchical clustering was performed with SRPLOT (accessed on 1 September 2023 at https://www.bioinformatics.com.cn).

Thrombospondin 1 is a counter-adhesive ECM protein, which is also found in platelets. It plays many roles and was detected by proteomics in the exudate from *B. asper* venom-injected mice [19]. Thrombospondin 1 is known to bind collagens [26] and to activate TGFβ [27], which is a pleiotropic cytokine that, among its many actions, promotes fibrosis [28]. The Nanostring analysis of the TGFβ pathway revealed up-regulation of the genes coding for TGFβ and TGFβ receptor at some time intervals, as well as LRRC 32, a key regulator of TGFβ (Figure 4 and Figure 5) [29]. The enzyme lysyl oxidase was also up-regulated. It is related to the metabolism of collagen and has been described in a variety of fibrotic diseases [30]. Taken together, these observations highlight a scenario of up-regulation of fibrosis-promoting genes. 

We also investigated gene expression related to myofibroblast regulation since these cells play a key role in wound contraction but may also stimulate fibrosis in a variety of pathologies [31]. The genes coding for chemokine interferon-γ-inducible protein-10 (CXCL10), integrin subunit α5, and lysyl oxidase, and connective tissue growth factor (CTGF), all related to the myofibroblast regulation pathways, were up-regulated in tissue affected by the two venoms (Figure 2 and Figure 3), reflecting stimuli for myofibroblast proliferation and collagen production. Our findings agree with previous studies on muscle repair and regeneration after myonecrosis induced by *B. asper* venom showing a deficient regeneration associated with increased fibrosis, which is very likely a consequence of fibroblast proliferation and fibrillar collagen synthesis [32,33,34]. Thus, our data provide clues to understanding the imbalance between myoblast and fibroblast proliferation in muscle tissue affected by viperid venoms, which may shed light on possible ways to modulate this imbalance to favor muscle regeneration instead of fibrosis.

### 3.3. Innate Immune System

Gene expression related to the pathways of NF-κB, cytokine signaling, and Toll-like receptors were evaluated. A variable pattern of up-regulation was observed in many genes in tissue samples from mice injected with these venoms, although there was a higher expression of several genes associated with these pathways in the case of *B. asper* venom. The highest level of most of these transcripts in the case of *B. asper* was observed at 6 h (Figure 4 and Figure 5). Some up-regulated genes from these pathways with the two venoms included IL1β, IL1R1, CD14, Myd88, TGFβ, TGFβR, and the chemokines CCL2 and CXCL10. (Figure 4 and Figure 5).

Up-regulation of IL1β agrees with previous observations of elevated levels of this cytokine in muscle tissue and peritoneal fluid lavage from mice injected with several *Bothrops* sp venoms, including *B. asper* [35,36,37]. IL1β is a pleiotropic cytokine with a variety of proinflammatory effects, including neutrophil, T cell, B cell, and endothelial cell activation, monocyte differentiation, and TH17 response, by high expression of IL1β in macrophages and neutrophils inducing an increase in CD45 cells infiltration [38,39]. In chronic inflammation, IL1β is related to angiogenesis and immunosuppression of macrophages characterized by the expression of M2 macrophages [40]. Interestingly, IL1β is also involved in wound healing delay, promotes fibrosis, and impairs re-epithelialization [39,41].

CCL2 (monocyte chemoattractant protein-1, MCP-1) is a potent chemoattractant for monocytes/macrophages but also attracts other cells such as T cells, B cells, NK cells, and DC cells [42]. In addition to its chemoattractant effect, it impacts leukocyte function, induces autophagy, macrophage polarization according to the context of the microenvironment, and cytokine secretion, including TNF-α, IL1β, IL6, IL10, and IL12 [42,43,44]. CXCL10 mediates the activation and recruitment of leukocytes such as T cells, monocytes, and NK cells. It is secreted by several cells in response to IFN-γ. CXCL10 has pleiotropic effects, including inflammatory process, inhibition of neovascularization in early wound healing, and immune fibroblast activation [45,46]. Overall, the observed patterns of expression of cytokine and chemokine genes underscore an inflammatory landscape associated with leukocyte infiltration, activation of immune cells, synthesis of a plethora of mediators, and other inflammatory events, in agreement with the pro-inflammatory action of viperid snake venoms [6]. 

It is of little surprise that we identified the overexpression of myeloid cell markers CD14 and Myd88. The former is a membrane-associated protein that binds the complex of bacterial lipopolysaccharide (LPS) and LPS-binding protein and is expressed in monocytes, macrophages, and dendritic cells [47,48]. Myd88 is an adapter in immune cells that has a pivotal role in innate immunity through Toll-like receptors, acting as an adaptor molecule that relays signals from outside the cell to intracellular proteins [47,49].

Innate immunity is likely to play a role in the inflammation in tissues affected by snake venom toxins by detecting molecular patterns associated with venom components, i.e., venom-associated molecular patterns (VAMPs) [50], and with fragments of cells and extracellular matrix, i.e., damage-associated molecular patterns (DAMPs) released as a consequence of the tissue-damaging activity of venoms [7]. It was, therefore, of interest to assess the expression of genes related to TLR signaling. The observed up-regulation of TLR4 in the case of *B. asper* venom at 6 h (Figure 4) is relevant as this receptor has been associated with inflammation induced by exudates collected from tissue affected by *B. asper* venom [7] and by other venoms and toxins [51,52]. Thus, the up-regulation of genes related to TLR signaling may reflect an ongoing process of innate immune system response to the action of these venoms in muscle tissue. Noteworthy, there were differences in the expression of these genes between tissues injected with these venoms (Figure 5). Such differences might be related to previously described variations in the inflammatory response after injection of these two venoms in mice [16].

### 3.4. Programmed Cell Death and Autophagy

**Figure 6:**
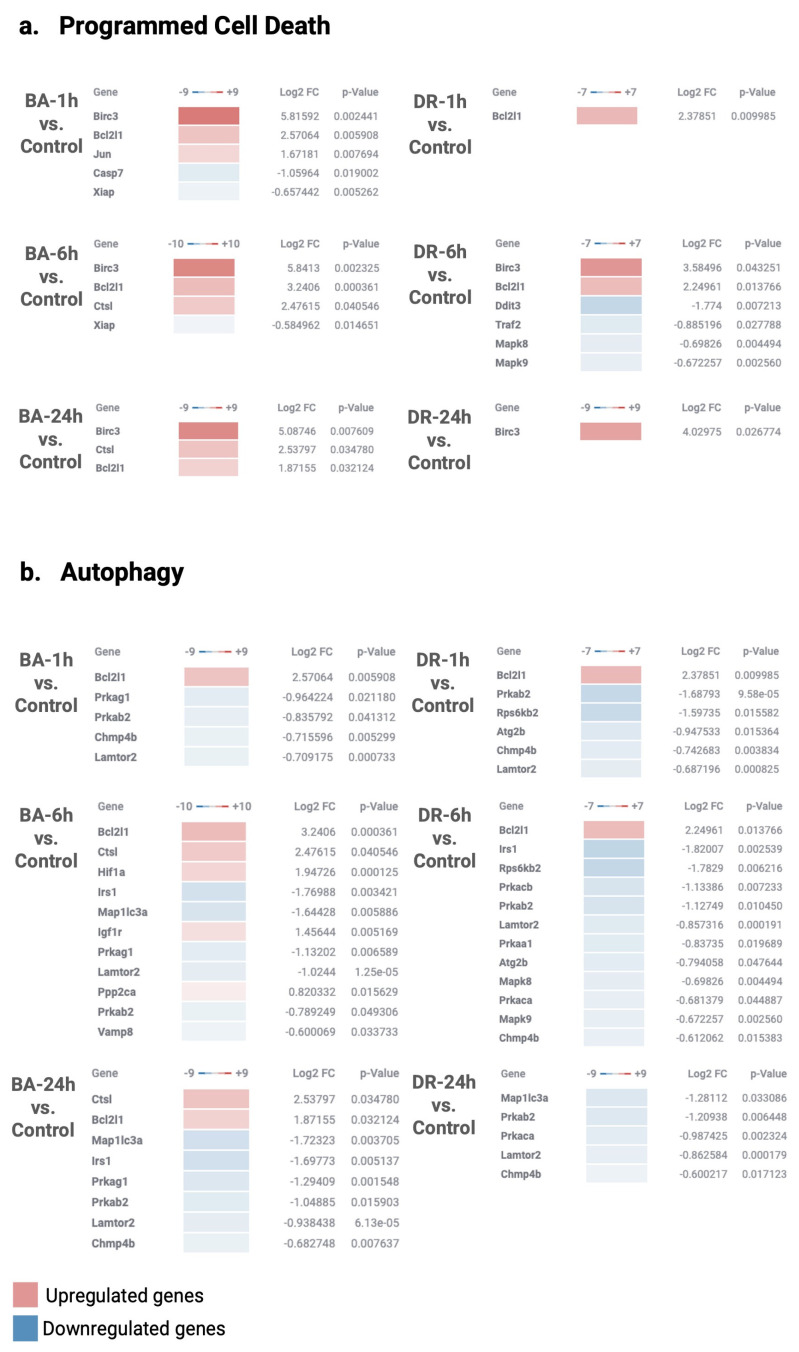
NanoString GSA pathway analysis of programmed cell death and autophagy gene alterations in muscle tissue injected with venoms of *B. asper* and *D. russelii*, as compared to controls injected with PBS. NanoString GSA pathway analysis. The Directed Global Significance scores are represented by up-regulated (red) and down-regulated (blue) genes at different time points in relation to the control samples. The *p*-value and Log2 FoldChange are shown to the right. Global Significance Score > 1.0. Graphic created in the Rosalind software (https://rosalind.bio/ version 3.38.6.1, accessed on 5 March 2023).

**Figure 7:**
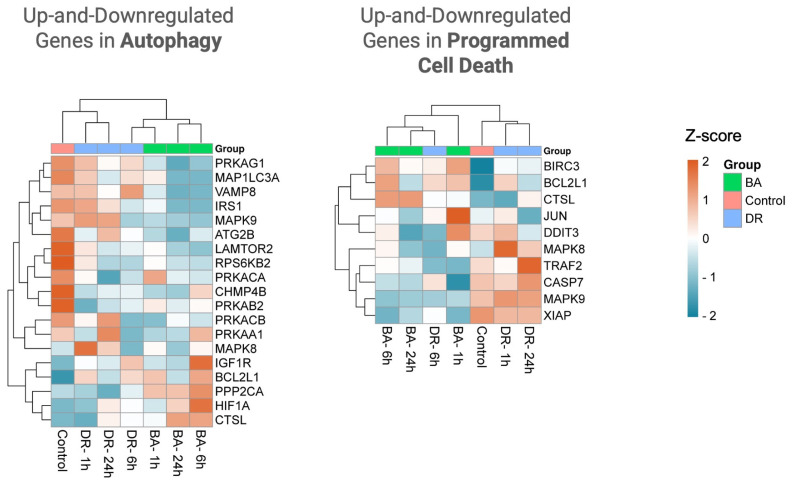
Heat maps of the programmed cell death and autophagy gene alterations in samples from mice injected with either PBS (control), *B. asper,* or *D. russelii* venoms. The heat maps were made using Log2 of transcript counts of the up-and down-regulated significant genes in each pathway using the Gene Set Analysis (GSA). Each row represents the gene monitoring over time after venom injection. Euclidean distance and complete linkage methods were applied. Hierarchical clustering is performed with SRPLOT (accessed on 1 September 2023 at https://www.bioinformatics.com.cn).

Genes associated with programmed cell death and autophagy were also analyzed (Figure 6 and Figure 7), as it is likely that these phenomena develop in tissue affected by snake venoms and toxins [34,53]. *Bothrops* sp. venom components have been shown to induce apoptosis and autophagy of various cell types in culture [54,55,56,57]. The genes BIRC3 and BCL2L1 were up-regulated in tissue injected with the two venoms. These genes are associated with programmed cell death. BIRC3 encodes a member of inhibitors of apoptosis (IAP) proteins that inhibit apoptosis by binding to TNFα-associated factors TRAF1 and TRAF2 and interfere with the activation of caspases [58]. BCL2L1 encodes for a protein of the BCL-2 family. These proteins are located in the mitochondrial outer membrane, regulating its permeabilization, and members of this family of proteins exert pro- and anti-apoptotic functions. Moreover, XIAP, an inhibitor of apoptosis [59], is down-regulated in the case of *B. asper* venom. In tissue injected with this venom, there is down-regulation of CASP7, a proapoptotic caspase (Figure 7). The observed pattern of expression of programmed cell death-related genes in our model calls for further studies aimed at understanding their role in venom-induced pathology. 

Little is known about the role of autophagy in tissues affected by snake venoms. Autophagy plays a key role in tissue homeostasis and is regulated by an abundant set of genes [60]. In vitro, some snake toxins have been described to induce autophagy [56], but whether this phenomenon occurs in vivo in experimental envenoming is largely unknown. With some exceptions, most genes associated with autophagy were down-regulated with the two venoms, although a few were up-regulated, with quantitative differences between venoms (Figure 6 and Figure 7). Among the genes associated with autophagy and up-regulated in muscle injected with *B. asper* venom are CTSL and PPP2CA (Figure 7). The former encodes cathepsin L, a lysosomal cysteine proteinase that has been associated with several pathologies and is responsible for myofibrillar degradation in myopathies [61]. PPP2CA gene encodes the protein phosphatase 2 catalytic subunit and is implicated in the negative control of cell growth and division [62]. 

The gene LAMTOR2 is down-regulated in tissue injected with both venoms. It codes for a member of the Ragulator/LAMTOR complex known to regulate mTOR and has a role as a late endosomal/lysosomal adaptor [63,64]. Deficiencies in this gene affect innate and adaptive immunity, having an impact on endosomal and lysosomal biogenesis [63]. The gene IGF1R, a receptor of insulin-like growth factor, is up-regulated at 6 h in both venoms (Figure 7). Its inhibition is known to reduce autophagy [65], thus, its expression would stimulate this cell death process. The gene VAMP8 is down-regulated in tissue injected with *B. asper* venom. It codes for a SNARE protein located in the lysosomal membrane that plays a key role in the fusion of lysosomes with autophagosomes [66]. On the other hand, the genes ATG2B and IRS1 are down-regulated in tissue affected by both venoms, especially in the case of *B. asper* (Figure 7). The former is related to the autophagic flow [67]. Moreover, several genes related to the subunits of AMP-activated protein kinase, i.e., PRKAG1, PRKACA, PRKAB2, are down-regulated in tissue affected by the venoms. These genes are involved in responses to various forms of cellular stress and are positive regulators of autophagy [68]. Taken together, our findings reveal a trend toward an expression pattern that results in the inhibition of autophagy. Since this process is an adaptation of cells to various forms of stress, its inhibition may have implications for the overall development of venom-induced tissue damage and repair.

## 4. Conclusions

Our findings reveal a complex and intricate pattern of gene expression in muscle injected with *B. asper* and *D. russelii* venoms, with similarities and differences between the venoms and variations along the time course of the experiment. Rapid up-regulation and down-regulation of many genes occurred starting in the first hour after the onset of envenoming, underscoring a rapid response of muscle tissue to the venoms. We focused our analysis on genes related to ECM synthesis and degradation and myofibroblast regulation, as well as on genes related to the innate immune response, programmed cell death, and autophagy. Up-regulated genes included various genes related to collagen metabolism and myofibroblast proliferation, probably associated with the onset of fibrosis and tissue repair. A variety of genes related to the immune system were also up-regulated, suggesting activation of innate immune pathways as a consequence of tissue-damaging effects of the venoms. Moreover, alterations also occurred in genes related to programmed cell death and autophagy, underscoring that these processes may be involved in venom-induced tissue damage and repair. In particular, our findings on the expression of genes related to autophagy provide novel clues on this hitherto unexplored aspect of venom-induced pathology. These findings pave the way for future experiments in which drugs that modulate apoptosis and autophagy can be used to assess the effects of these cell death pathways in the overall outcome of venom-induced tissue damage. Further studies are needed to link these acute gene expression events with the development of medium- and long-term phenomena taking place in the tissues, associated with repair, regeneration, and chronic inflammation in snakebite envenoming. 

## Data Availability

Data are available in the GeoAccession number GSE248215 at: https://www.ncbi.nlm.nih.gov/geo/query/acc.cgi?acc=GSE248215, accessed on 1 September 2023.

## Supplementary Materials

The following supporting information can be downloaded at https://www.mdpi.com/article/10.3390/biom14030278/s1. Figure S1. Quality Control and Normalized Data. (A) Principal Component Analysis (PCA) showed a clear separation of venom toxins time point group can be seen in the PC1 vs. PC2 results, meaning that changes in this variable cause clear, consistent changes in the data. The analysis was made using https://www.bioinformatics.com.cn/srplot, accessed on 23 September 2023 (B–D) The quality control of samples was verified using spike-ins of positive and negative probes, and housekeeping expression levels. The graphics were generated using the Rosalind cloud platform. Table S1. Mouse Fibrosis Gene List and Probe Annotation. Table S2. Raw Counts and Log2 Normalized Counts. Data acquisition is carried out in the NanoString nCounter Digital Analyzer with the ‘Max’ Field of View (FOV) setting to 555 images per sample in a 6 h scan per cartridge. Raw counts were normalized using the positive controls, and target genes were normalized to the five internal reference genes. Table S3. Differential analysis—The gene expression was analyzed by comparing each time point of venom injection to the control group (PBS). Differential gene expression analysis was calculated using the generalized linear model (GLM) assuming a negative binomial distribution. Table S4. GSA Pathway Analysis. The enrichment pathways were analyzed using the Gene Set Analysis (GSA) module from NanoString on the Rosalind cloud platform.
