# Supplementary figures and images for: A Complex Pattern of Gene Expression in Tissue Affected by Viperid Snake Envenoming: The Emerging Role of Autophagy-Related Genes
Source: Biomolecules. 2024 Feb 26;14(3):278. doi: 10.3390/biom14030278 (PMC10967929; doi:10.3390/biom14030278)

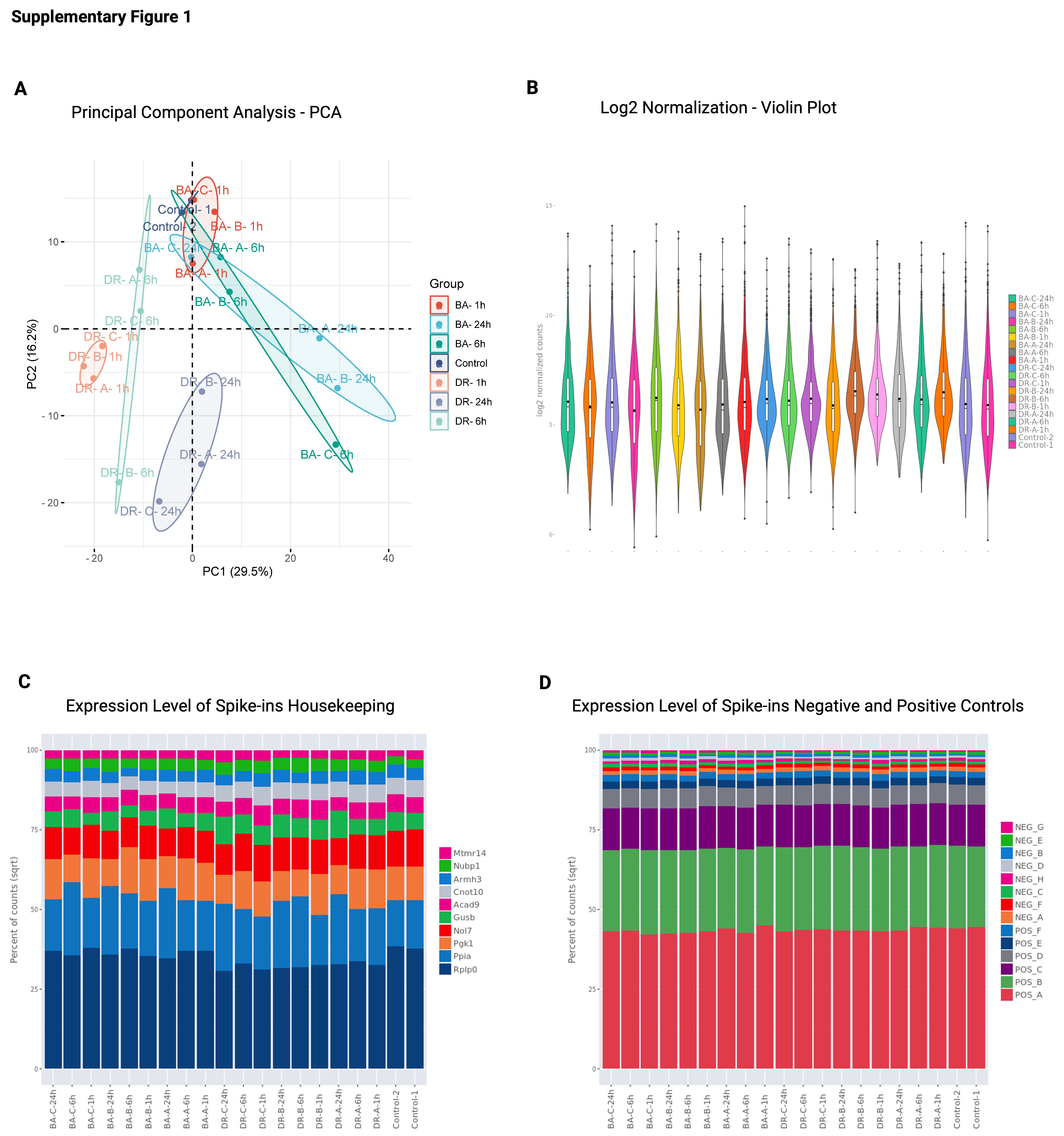

Supplement: Supplementary file 1 [file biomolecules-14-00278-s001.zip › Supplementary Figure S1.jpg]
